# Integrative Proteomics and Machine Learning Identify SLC27A2 as a Candidate Biomarker and Potential Mediator of Pyrotinib Response in HER2-Positive Breast Cancer

**DOI:** 10.3390/cancers18162702

**Published:** 2026-08-20

**Authors:** Shiyu Zhang, Xiaolu Yang, Yujia Zhang, Siqi Cheng, Haoyang Niu, Xiaomei Liao, Yilun Li, Li Ma

**Affiliations:** 1 Department of Breast Disease Center, The Fourth Hospital of Hebei Medical University, No. 12, Jiankang Road, Chang’an District, Shijiazhuang 050010, China; 2 Department of Breast Surgery, Xingtai People’s Hospital, Xingtai 054031, China; 3 Department of Pain and Rehabilitation, The Fourth Hospital of Hebei Medical University, Shijiazhuang 050010, China

**Keywords:** HER2-positive breast cancer, pyrotinib, SLC27A2, fatty acid metabolism, biomarker

## Abstract

**Background**: Pyrotinib, an irreversible pan-HER tyrosine kinase inhibitor, has demonstrated substantial clinical efficacy in patients with HER2-positive breast cancer (BC). However, intrinsic and acquired resistance remain important challenges limiting therapeutic benefit, and reliable biomarkers for predicting pyrotinib response are currently unavailable. This study aimed to identify molecular determinants associated with pyrotinib resistance and uncover their underlying mechanisms. **Methods**: Pre-treatment tumour samples from an exploratory discovery cohort of 12 patients with HER2-positive BC receiving pyrotinib-containing neoadjuvant therapy were analysed by proteomic profiling. Differentially expressed proteins (DEPs) between the pathological complete response (pCR) and non-pCR groups were integrated with weighted gene co-expression network analysis and protein–protein interaction network analysis to identify candidate proteins. The prognostic relevance of the candidate genes was subsequently evaluated using 127 machine-learning strategies across three independent BC cohorts. Models were ranked according to the mean area under the receiver operating characteristic curve (AUC) across two evaluation cohorts, and SHapley Additive exPlanations (SHAP) analysis was performed separately in both cohorts to prioritise a candidate for subsequent investigation. In vitro and in vivo experiments were then conducted to evaluate the biological role of the prioritised candidate and its association with pyrotinib sensitivity. Finally, the association between pre-treatment SLC27A2 expression and pCR was evaluated in an independent, non-overlapping retrospective cohort of 103 patients receiving pyrotinib-containing neoadjuvant therapy. **Results**: Exploratory proteomic profiling of 12 pre-treatment tumour samples identified 617 DEPs between the pCR and non-pCR groups. Among 127 machine-learning strategies used to evaluate the prognostic relevance of the candidate genes, the glmBoost–random forest model achieved the highest mean AUC across the two evaluation cohorts (mean AUC = 0.678). SHAP analysis showed that *SLC27A2* ranked second in GSE16446 and first in GSE48390 according to mean absolute SHAP values, supporting its prioritisation for subsequent investigation. Functional experiments showed that *SLC27A2* promoted proliferation, migration, invasion, and epithelial–mesenchymal transition in HER2-positive BC cells. *SLC27A2* knockdown enhanced pyrotinib sensitivity in vitro. In the xenograft experiment using female BALB/c nude mice, both SLC27A2 knockdown and pyrotinib treatment reduced tumour growth, and a significant interaction between the two factors was observed for endpoint tumour weight (*p* for interaction = 0.041). Mechanistically, SLC27A2 knockdown reduced lipid accumulation and PPARα expression, whereas pharmacological activation of PPARα partially attenuated the increase in pyrotinib sensitivity induced by SLC27A2 knockdown. Clinical validation further showed that high-pre-treatment SLC27A2 expression was independently associated with a lower likelihood of achieving pCR after pyrotinib-containing neoadjuvant therapy (OR = 0.10, 95% CI: 0.03–0.31, *p* < 0.001). **Conclusions**: SLC27A2 is a candidate factor associated with BC prognosis and reduced pyrotinib sensitivity in HER2-positive BC. Preclinical findings suggested that PPARα-related fatty acid metabolism may contribute to the association between SLC27A2 and pyrotinib response, while clinical validation showed that high-pre-treatment SLC27A2 expression was independently associated with a lower likelihood of achieving pCR following pyrotinib-containing neoadjuvant therapy. These findings support SLC27A2 as a candidate response-associated biomarker and potential therapeutic target, although further mechanistic investigation and external clinical validation are required.

## Simple Summary

Patients with HER2-positive breast cancer can benefit from pyrotinib-containing treatment, but therapeutic responses vary considerably, and reliable biomarkers for predicting treatment efficacy remain limited. In this study, we integrated proteomic profiling, machine learning, SHapley Additive exPlanations analysis, and functional experiments to identify SLC27A2 as a candidate factor associated with both pyrotinib sensitivity and breast cancer prognosis. Functional experiments showed that SLC27A2 promoted malignant phenotypes in HER2-positive breast cancer cells, whereas its knockdown increased pyrotinib sensitivity in vitro and suppressed xenograft growth in vivo. In the animal model, SLC27A2 knockdown showed a significant interaction with pyrotinib treatment for endpoint tumour weight. Our findings further suggested that PPARα-related fatty acid metabolism may contribute to this association. In addition, high SLC27A2 expression in pre-treatment tumour samples was associated with a lower likelihood of achieving pathological complete response after pyrotinib-containing neoadjuvant therapy. Collectively, these findings support SLC27A2 as a potential response-associated biomarker and therapeutic target in HER2-positive breast cancer.

## 1. Introduction

Breast cancer (BC) is the most prevalent malignancy among women globally [1]. Approximately 15–20% of BC cases exhibit human epidermal growth factor receptor 2 (HER2) overexpression or gene amplification, defining the HER2-positive subtype. This subtype is characterised by aggressive biological behaviour and poor clinical outcomes if untreated with anti-HER2 therapies [2,3,4].

Anti-HER2-targeted therapies play a pivotal role in delaying disease progression in patients with HER2-positive BC [5]. These therapies in clinical use can be categorised into three main groups: monoclonal antibodies (e.g., trastuzumab, pertuzumab), small-molecule tyrosine kinase inhibitors (TKIs; e.g., pyrotinib, lapatinib, tucatinib), and antibody–drug conjugates (ADCs; e.g., ado-trastuzumab emtansine, trastuzumab deruxtecan) [2]. Research suggests that combining two anti-HER2 agents with distinct or complementary mechanisms is more effective in blocking the HER2 pathway than monotherapy [6].

Pyrotinib is an irreversible pan-HER TKI that effectively inhibits the tyrosine kinase activity of epidermal growth factor receptor (EGFR), HER2, and HER4 receptors. Due to its irreversible binding properties, pyrotinib has a stronger inhibitory effect on the HER2 pathway compared to reversible TKIs, translating into superior anti-tumour effects [6,7]. In the PHILA study, first-line treatment with pyrotinib combined with trastuzumab and docetaxel extended progression-free survival (PFS) to 24.3 months in patients with HER2-positive recurrent or metastatic BC, outperforming trastuzumab plus docetaxel alone [6]. Similarly, the PHEDRA study demonstrated that pyrotinib combined with trastuzumab and docetaxel nearly doubled the pathological complete response (pCR) rate (41% vs. 22%) during neoadjuvant treatment for HER2-positive early-stage BC compared to a control group using placebo with trastuzumab and docetaxel [8]. Consistently, our prior multicenter retrospective study found that neoadjuvant therapy involving pyrotinib, trastuzumab, and chemotherapy achieved a significantly higher pCR rate compared to trastuzumab with chemotherapy alone (46.5% vs. 31.5%) [9]. These encouraging clinical results confirm pyrotinib’s status as an effective HER2-targeted drug and provide strong evidence for its wider clinical use in HER2-positive BC.

However, despite the significant clinical efficacy of pyrotinib, primary and acquired resistance remain the main challenges limiting its long-term therapeutic benefits. As we found in our previous retrospective study, a considerable proportion of patients failed to achieve pCR during the neoadjuvant phase, and these patients are more prone to disease recurrence during follow-up. These observations highlight the considerable interpatient heterogeneity in response to pyrotinib, suggesting that intrinsic molecular differences may influence therapeutic sensitivity. Therefore, elucidating the molecular mechanisms underlying pyrotinib resistance is essential for improving treatment efficacy, identifying novel therapeutic targets, and ultimately optimising the clinical management of patients with HER2-positive BC.

In this study, we performed exploratory proteomic profiling of pre-treatment biopsy samples from patients with HER2-positive BC who achieved pCR or had residual disease following pyrotinib-containing neoadjuvant therapy. Our objective was to identify candidate proteins associated with pathological response and to investigate their potential roles in pyrotinib sensitivity. The findings of this study are expected to provide new insights into the molecular basis of pyrotinib resistance and may facilitate the development of more effective therapeutic strategies for HER2-positive BC.

## 2. Materials and Methods

### 2.1. Proteomic Discovery Cohort and Proteomic Profiling

Pre-treatment biopsy samples were obtained from 12 patients with HER2-positive BC who received pyrotinib-containing neoadjuvant therapy at the Breast Disease Centre of the Fourth Hospital of Hebei Medical University. Samples were selected according to postoperative pathological response using a balanced 1:1 design, including six patients who achieved pCR and six patients with residual disease (non-pCR). Only samples containing sufficient tumour tissue and meeting the tissue-quality requirements for proteomic profiling were included. Proteomic profiling was performed by Shanghai Meiji Biomedical Technology Co., Ltd. (Shanghai, China). Each patient sample represented an independent biological replicate. Differentially expressed proteins (DEPs) between the pCR and non-pCR groups were identified using thresholds of |log_2_ fold change| ≥ 1.5 and adjusted *p* < 0.05.

#### Retrospective Clinical Cohort and Data Collection

An independent retrospective cohort of patients with HER2-positive BC who received pyrotinib-containing neoadjuvant therapy at the Breast Disease Centre of the Fourth Hospital of Hebei Medical University between January 2023 and December 2023 was included to evaluate the association between pre-treatment SLC27A2 expression and pathological response. The inclusion criteria were as follows: (1) pathologically confirmed HER2-positive invasive BC; (2) receipt of pyrotinib-containing neoadjuvant therapy; (3) completion of surgery following neoadjuvant therapy with an available postoperative pathological response assessment; and (4) availability of sufficient pre-treatment biopsy tissue for SLC27A2 immunohistochemical (IHC) staining. Patients were excluded if they had distant metastasis at diagnosis, had received previous systemic or anti-HER2 therapy for the current disease before initiation of neoadjuvant therapy, had insufficient tissue samples, or had incomplete clinicopathological data.

A total of 103 patients were included and classified into pCR and non-pCR groups according to postoperative pathological evaluation. pCR was defined as the absence of residual invasive carcinoma in both the breast and axillary lymph nodes, irrespective of the presence of residual ductal carcinoma in situ. None of these patients had been included in the proteomic discovery cohort. Clinicopathological characteristics, including age, menopausal status, tumour size, lymph node involvement, American Joint Committee on Cancer (AJCC) stage [10], oestrogen receptor (ER) status, progesterone receptor (PR) status, Ki-67 expression, and tumour-infiltrating lymphocytes (TILs), were collected from the medical records.

### 2.2. Functional Enrichment Analysis

GO and KEGG enrichment analyses were performed to explore the biological functions and pathways associated with the identified DEPs. Terms with significant enrichment were determined based on adjusted *p* values and were visualised using R software (v 4.5.1).

### 2.3. Weighted Co-Expression Network Analysis (WGCNA)

An exploratory WGCNA was performed using the normalised protein-abundance matrix from the 12-sample proteomic discovery cohort. Proteins with a standard deviation of ≤0.5 across samples were excluded. Data quality was assessed using the goodSamplesGenes function, followed by average-linkage hierarchical clustering based on Euclidean distance to identify potential sample outliers. No obvious outlier samples were detected, and all 12 samples were retained for analysis. Candidate soft-thresholding powers from one to 20 were evaluated, and a power of five was selected according to the scale-free topology criterion. An unsigned adjacency matrix was constructed and transformed into a topological overlap matrix. Modules were identified using dynamic tree cutting with a minimum module size of 50 and deepSplit = 2, followed by the merging of modules with an eigengene dissimilarity of <0.25. Pearson correlations between module eigengenes and pathological-response status were calculated, and the corresponding *p* values were determined using a Student’s *t*-based test. Given the limited size of the discovery cohort, WGCNA was used as an exploratory candidate-prioritisation approach. Proteins in the module showing the strongest association with non-pCR were intersected with the DEPs for subsequent candidate screening.

### 2.4. Protein–Protein Interaction (PPI) Network Construction and Candidate Target Identification

Candidate proteins were identified by intersecting proteins from the most significant WGCNA module with DEPs. A PPI network for the candidate proteins was then constructed using the STRING database and visualised with Cytoscape (v 3.9.1). Topological analysis of the network (assessing degree, closeness centrality, and clustering coefficient) was used to identify hub proteins, which were selected as candidate targets for further investigation.

### 2.5. GEO Data Preprocessing

Processed Series Matrix files were obtained from the Gene Expression Omnibus (GEO) database, and probes were mapped to gene symbols using the corresponding platform annotation files. Probes without valid or unambiguous gene-symbol annotations were removed, and the mean expression value was calculated when multiple probes mapped to the same gene. Missing probe-level values were omitted during aggregation, with no additional imputation performed. Before machine-learning analysis, gene expression values were Z-score standardised separately within each cohort. Because the development and evaluation cohorts were analysed separately, no cross-cohort batch-effect correction was applied.

### 2.6. Machine-Learning Modelling and SHAP Analysis

To explore the prognostic relevance of the core genes, 127 candidate machine-learning strategies combining feature-selection and binary-classification algorithms were evaluated using all-cause mortality status as the outcome. GSE20685 was used as the development cohort, whereas GSE16446 and GSE48390 were used as independent evaluation cohorts. Algorithm-specific internal cross-validation and tuning procedures were applied in the development cohort according to the implementation of each machine-learning algorithm. For multi-component strategies, the preceding algorithm or algorithms were used for feature selection, and the final algorithm was used for model construction. Strategies retaining fewer than three features after modelling were excluded from performance ranking. Models were ranked according to the mean AUC across the two evaluation cohorts; the AUC in the development cohort was displayed but was not used for model ranking. The 95% confidence interval for the AUC in each cohort was calculated using the DeLong method. Class imbalance was not modified by resampling. The complete list of algorithm combinations, cross-validation procedures, principal tuning settings, selected features, analysis status, cohort-specific AUCs with 95% confidence intervals, and mean evaluation AUCs is provided in Supplementary Table S1. SHAP analysis was subsequently applied to the highest-ranked model separately in GSE16446 and GSE48390. Global feature importance was quantified using the mean absolute SHAP value in each cohort.

### 2.7. Expression Analysis of SLC27A2 Using TCGA and GTEx Datasets

Following its prioritisation through machine-learning and SHAP analyses, the mRNA expression pattern of *SLC27A2* was further characterised using data from The Cancer Genome Atlas (TCGA) and Genotype-Tissue Expression (GTEx) projects. Pan-cancer comparisons were performed to evaluate differences in *SLC27A2* expression between tumour and normal tissues across multiple cancer types. *SLC27A2* expression was subsequently examined specifically in BC using the TCGA-BRCA cohort, including both unpaired comparisons between tumour and normal breast tissues and paired comparisons between tumour tissues and matched adjacent normal tissues. Expression values were presented as log_2_(TPM + 1). Unpaired comparisons were performed using the Wilcoxon rank-sum test, whereas paired tumour–normal comparisons were conducted using the Wilcoxon signed-rank test.

### 2.8. Cell Culture and Transfection

Cytological experiments were conducted using the two HER2-positive cell lines, SKBR3 and MDA-MB-453. Both cell lines were purchased from Wuhan Pricella Biotechnology Co., Ltd. (Wuhan, China) and cultured in a 37 °C incubator containing 5% CO_2_ using the specialised culture medium provided by the company.

For *SLC27A2* overexpression, a plasmid-encoding human *SLC27A2* was purchased from GenePharma Co., Ltd. (Suzhou, China) and transfected into cells according to the manufacturer’s instructions. For gene knockdown, small interfering RNA (siRNA) targeting *SLC27A2* (GenePharma, Suzhou, China) was transfected into cells following the manufacturer’s instructions.

### 2.9. Cell Proliferation Assay

To determine whether *SLC27A2* affects cellular growth, a CCK-8 assay was performed. After transfection, cells were distributed into 96-well plates (3000 cells/well). At indicated time points (0, 24, 48, and 72 h after seeding), CCK-8 solution was added, and cell viability was quantified by measuring the optical density at 450 nm.

### 2.10. Colony Formation Assay

A colony formation assay was conducted to determine whether *SLC27A2* affects the long-term proliferative capacity of BC cells. Following transfection, cells were seeded into six-well plates (1 × 10^3^ cells/well) and cultured for 2 weeks to allow colony development. Colonies were then preserved by formaldehyde fixation and detected using crystal violet staining.

### 2.11. Migration and Invasion Assays

Wound-healing assays were employed to evaluate cell migration ability. When transfected cells reached a confluency of 80% or higher, a 1000 μL pipette tip was used to create scratch areas. After 48 h, photographs of the scratch areas were captured at the same locations to quantify cell migration. The migration area was calculated using the formula: (Initial scratch area − Scratch area at 48 h)/Initial scratch area × 100%.

In addition, Transwell assays were used to evaluate the effects of *SLC27A2* on cell migration and invasion. Separately, 50,000 transfected cells were seeded into the upper chamber of Transwell plates with or without matrix gel (the culture medium did not contain serum), and 650 μL of serum-containing culture medium was added to the lower chamber. After 48 h of culture, unmigrated cells were washed away with PBS, the bottom of the upper chamber was fixed with formaldehyde, and the cells were stained with crystal violet and counted.

### 2.12. Pyrotinib Sensitivity Assay

To assess sensitivity to pyrotinib, control cells and *SLC27A2*-knockdown cells were seeded at a density of 5000 cells per well in a 96-well plate and treated with pyrotinib at gradually increasing concentrations. After 48 h of culture, cell viability was measured using the CCK-8 assay, and cell viability curves were plotted.

### 2.13. Lipid Accumulation and Triglyceride Measurement

Intracellular lipid accumulation was assessed by Oil Red O staining. Briefly, cells were fixed with formaldehyde, stained with Oil Red O solution, and lipid droplet accumulation was visualised by light microscopy. Triglyceride levels were quantitatively measured using a commercial triglyceride assay kit (Nanjing Jiancheng Bioengineering Institute, Nanjing, China) according to the manufacturer’s instructions.

### 2.14. PPARα Rescue Experiment

To investigate whether PPARα activity contributes to the effects of SLC27A2 knockdown on pyrotinib sensitivity, *SLC27A2*-knockdown cells were treated with the selective PPARα agonist GW7647 (MedChemExpress, Monmouth Junction, NJ, USA). Changes in pyrotinib sensitivity were evaluated using CCK-8 assays.

### 2.15. Western Blotting

Total protein was extracted from cells using RIPA lysis buffer and quantified using a bicinchoninic acid assay. Equal amounts of protein (30 μg per lane) were separated by SDS–PAGE and transferred onto polyvinylidene fluoride membranes. After blocking, the membranes were incubated overnight at 4 °C with the corresponding primary antibodies. The membranes were subsequently incubated with the appropriate horseradish peroxidase-conjugated secondary antibodies. Protein bands were visualised using an enhanced chemiluminescence detection system, with β-actin used as the loading control. Detailed information on the primary and secondary antibodies, including manufacturers, catalogue numbers, and dilution ratios, is provided in Supplementary Table S2.

### 2.16. IHC Staining

Paraffin-embedded clinical and xenograft tumour tissues were sectioned at a thickness of 4 μm and subjected to IHC staining. Briefly, tissue sections were baked at 65 °C for 1 h, followed by deparaffinization, rehydration, antigen retrieval, blocking, overnight incubation with primary antibodies, incubation with secondary antibodies for 30 min, and visualisation using 3,3′-diaminobenzidine (DAB).

For each section, three randomly selected fields within the tumour area were captured using a microscope. The staining intensity of SLC27A2 was quantitatively analysed using Image-Pro Plus software (v 6.0). The mean optical density (MOD) was calculated as the integrated optical density (IOD) of positively stained areas divided by the corresponding tumour area. The average MOD value of all samples was used as the cutoff value to classify SLC27A2 expression levels. Samples with MOD values above the cutoff were defined as the high-SLC27A2-expression group, whereas those below the cutoff were classified as the low-SLC27A2-expression group [11].

### 2.17. Xenograft Mouse Model

A total of 24 female BALB/c nude mice aged 4–6 weeks and weighing 13–15 g were obtained from Beijing Vital River Laboratory Animal Technology Co., Ltd. (Beijing, China). The mice were housed under specific pathogen-free conditions at 20–26 °C and 40–70% relative humidity, with no more than five mice per cage, and acclimatised for 1 week. General health and humane-endpoint criteria were monitored every 3 days.

Based on preliminary xenograft data, six mice per group were required using endpoint tumour weight as the primary outcome, a two-sided α of 0.05, and 80% statistical power. Each mouse was considered an experimental unit. SKBR3 cells stably expressing shNC or shSLC27A2 were injected subcutaneously into the mammary fat pad (5 × 10^6^ cells/mouse). Mice were randomly allocated to four groups using a random-number table in a 2 × 2 factorial design: shNC plus vehicle, shSLC27A2 plus vehicle, shNC plus pyrotinib, and shSLC27A2 plus pyrotinib.

From day 21 to day 35, pyrotinib was administered once daily by oral gavage at 5 mg/kg, while vehicle groups received normal saline. Personnel responsible for treatment administration, tumour measurement, and data analysis were unaware of group allocation. Tumour dimensions and body weight were recorded every 5 days, and tumour volume was calculated as *V* = (*L* × *W*^2^)/2. Humane endpoints included a tumour volume of 1500 mm^3^, tumour-surface ulceration, body-weight loss exceeding 15%, or tumour burden exceeding 10% of normal body weight. No animals died, met the humane endpoints, or were excluded. On day 35, mice were euthanised by cervical dislocation, and tumours were collected for weighing and IHC analysis. All procedures were approved by the Institutional Animal Care and Use Committee of the Fourth Hospital of Hebei Medical University (No. IACUC-4th Hos Hebmu-20250112; 6 January 2025) and followed the ARRIVE 2.0 guidelines.

### 2.18. Statistical Analysis

Statistical analyses were performed using R software and GraphPad Prism (v 9.5). Unless otherwise stated, in vitro experiments were independently repeated three times, and data are presented as the mean ± SD. Technical replicates were averaged before statistical analysis. The number of biological replicates, definition of error bars, statistical test, and post hoc procedure used for each experiment are specified in the corresponding figure or table legend.

Multiple testing in proteomic and enrichment analyses was controlled using the Benjamini–Hochberg procedure. Clinicopathological associations were evaluated using appropriate categorical tests and binary logistic regression, with odds ratios and 95% confidence intervals reported.

Longitudinal tumour-volume and body-weight data were analysed using linear mixed-effects models, with mouse identity included as a random effect. Endpoint tumour weights were analysed using two-way ANOVA, with SLC27A2 knockdown, pyrotinib treatment, and their interaction included in the model. All tests were two-sided. *p* < 0.05 was considered statistically significant.

## 3. Results

### 3.1. Exploratory Identification of Proteins Associated with Response to Pyrotinib-Containing Neoadjuvant Therapy

**Figure 1:**
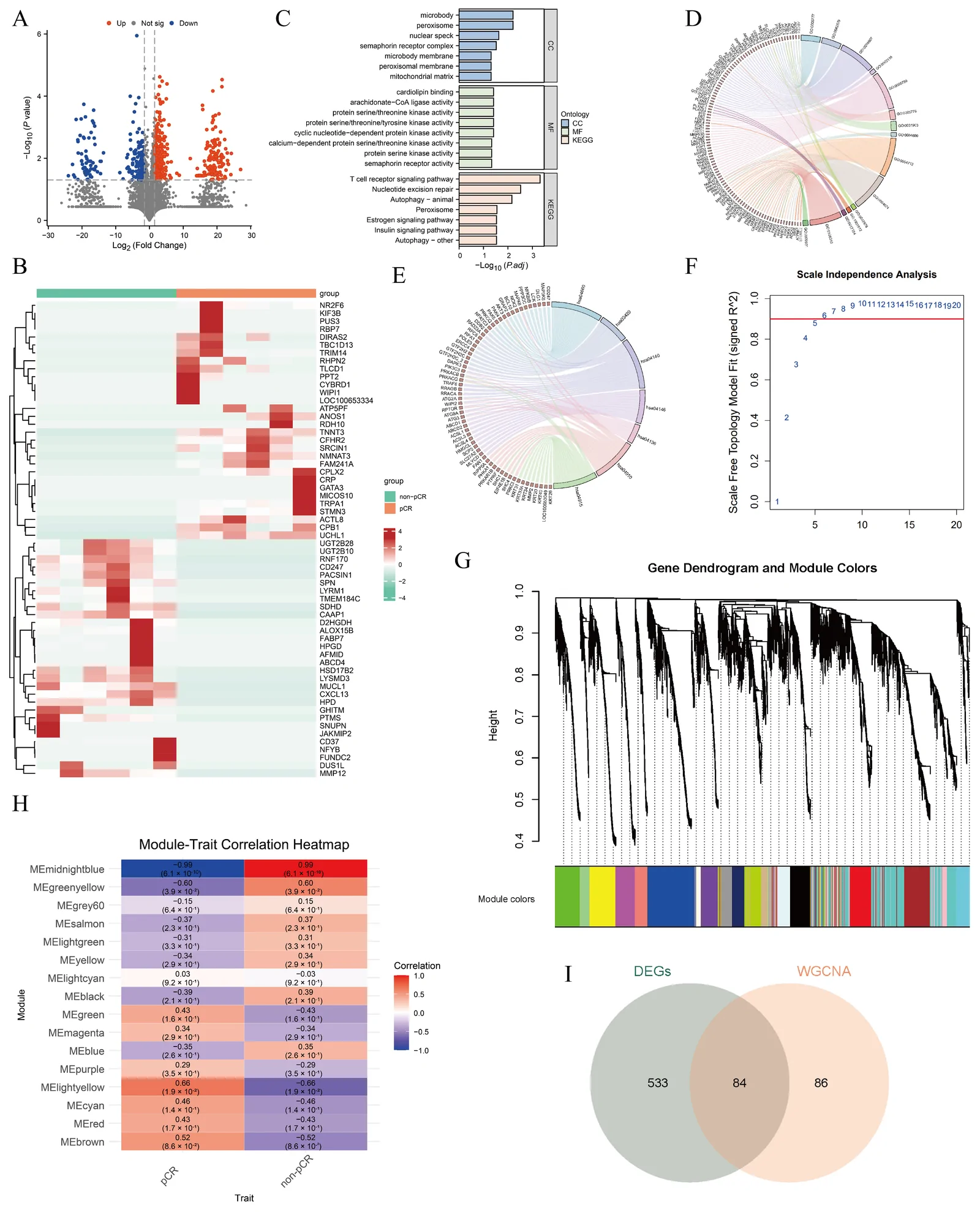
Exploratory proteomic and weighted co-expression network analyses identified candidate proteins associated with pathological response to pyrotinib-containing neoadjuvant therapy in HER2-positive breast cancer. (**A**) Volcano plot showing differentially expressed proteins (DEPs) between the pCR and non-pCR groups. DEPs were identified using thresholds of |log_2_ fold change| ≥ 1.5 and adjusted *p* < 0.05; red, blue, and grey dots indicate upregulated, downregulated, and non-significantly altered proteins, respectively. (**B**) Heatmap showing the expression patterns of representative DEPs across the six pCR and six non-pCR samples. (**C**) Gene Ontology (GO) and Kyoto Encyclopaedia of Genes and Genomes (KEGG) enrichment analyses of the identified DEPs. (**D**) Chord diagram showing the relationships between DEPs and significantly enriched GO terms. (**E**) Chord diagram showing the relationships between DEPs and significantly enriched KEGG pathways. (**F**) Scale-free topology fit and mean connectivity across candidate soft-thresholding powers from 1 to 20. A soft-thresholding power of 5 was selected for network construction. (**G**) Hierarchical clustering dendrogram of proteins and the corresponding module assignments obtained using dynamic tree cutting and module merging. Modules were detected using a minimum module size of 50 and were merged at an eigengene dissimilarity threshold of 0.25, resulting in 16 protein co-expression modules. (**H**) Heatmap showing Pearson correlations between module eigengenes and pathological-response status. Correlation coefficients and corresponding *p* values are displayed in each cell. The midnightblue module, comprising 170 proteins, showed the strongest observed association with pathological response and was positively correlated with non-pCR (*r* = 0.99, *p* = 6.1 × 10^−10^) and inversely correlated with pCR (*r* = −0.99, *p* = 6.1 × 10^−10^). (**I**) Venn diagram showing the intersection between the 617 DEPs and the 170 proteins in the midnightblue module, yielding 84 overlapping candidate proteins.DEP, differentially expressed protein; GO, Gene Ontology; HER2, human epidermal growth factor receptor 2; KEGG, Kyoto Encyclopaedia of Genes and Genomes; pCR, pathological complete response; WGCNA, weighted gene co-expression network analysis.

In the exploratory proteomic discovery cohort, pre-treatment tumour samples from six patients who achieved pCR and six patients with residual disease following pyrotinib-containing neoadjuvant therapy were analysed. Using thresholds of |log_2_ fold change| ≥ 1.5 and adjusted *p* < 0.05, we identified 617 differentially expressed proteins between the pCR and non-pCR groups, including 419 upregulated and 198 downregulated proteins (Figure 1A,B).

To characterise the biological functions of these DEPs, GO and KEGG enrichment analyses were subsequently performed. A total of 15 GO terms and seven KEGG pathways were significantly enriched (Figure 1C–E). The enriched GO terms were predominantly related to intracellular organelles (microbody, peroxisome, and mitochondrial matrix) and the activity of various protein kinases. KEGG enrichment analysis revealed that DEPs were significantly enriched in pathways associated with T cell receptor signalling, autophagy, hormone signalling and metabolic processes.

Exploratory WGCNA was subsequently performed to identify protein co-expression modules associated with pathological response to pyrotinib-containing neoadjuvant therapy. Sample-quality assessment and hierarchical clustering identified no obvious outliers among the 12 discovery samples, and all samples were retained for analysis. A soft-thresholding power of five was selected according to the scale-free topology criterion (Figure 1F), and 16 protein co-expression modules were identified (Figure 1G). The midnightblue module, comprising 170 proteins, showed the strongest observed association with pathological response. Its module eigengene was positively correlated with non-pCR (r = 0.99, *p* = 6.1 × 10^−10^) and inversely correlated with pCR (r = −0.99, *p* = 6.1 × 10^−10^; Figure 1H). Intersection of the 170 proteins in the midnightblue module with the 617 differentially expressed proteins identified 84 candidate proteins for subsequent analysis (Figure 1I).

### 3.2. Machine-Learning and SHAP Analyses Prioritise SLC27A2 for Further Investigation

**Figure 2:**
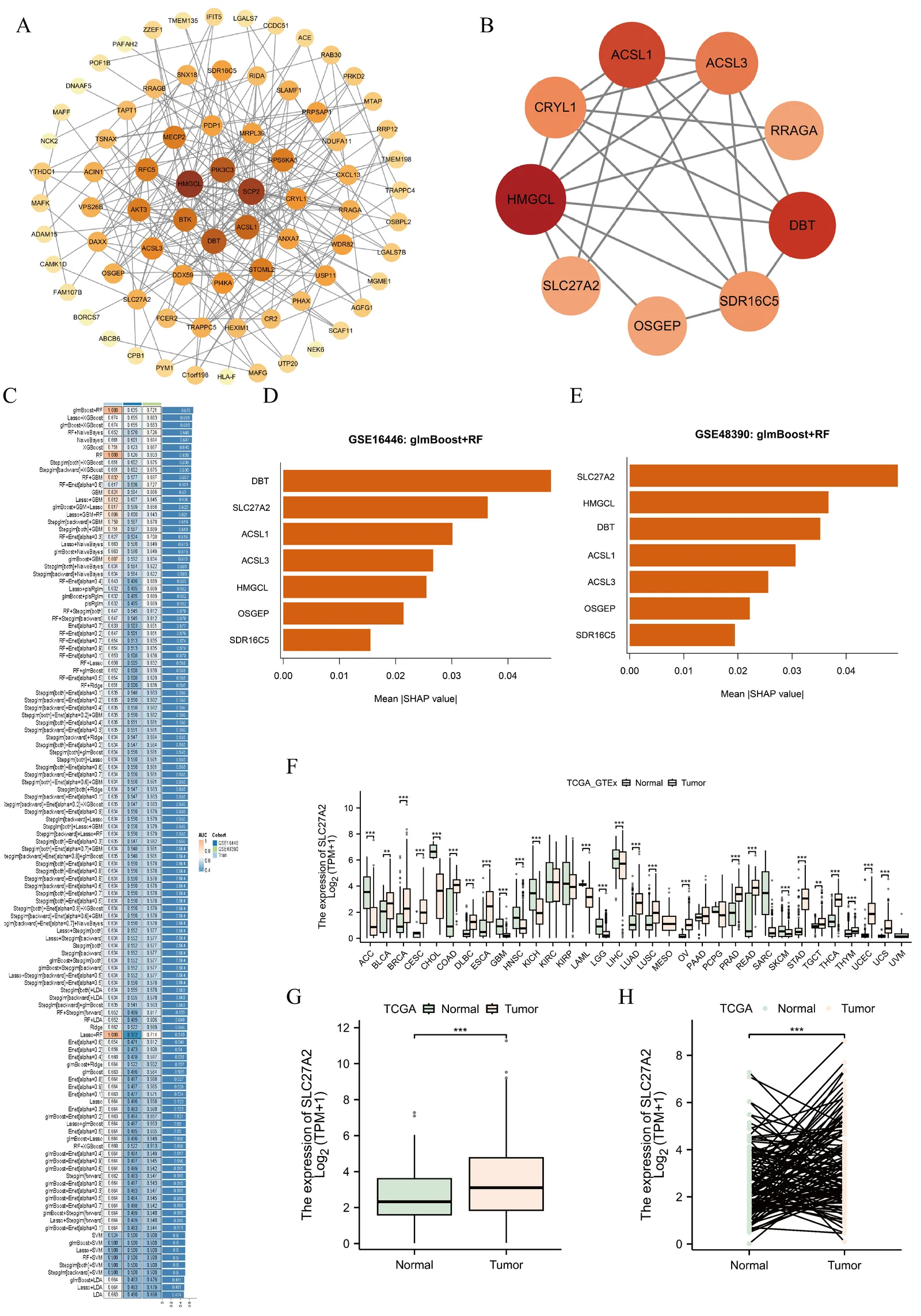
Integrative network, machine-learning, SHAP, and expression analyses prioritise SLC27A2 for further investigation. (**A**) Protein–protein interaction (PPI) network constructed from the 84 overlapping proteins identified by integrating the differentially expressed proteins with proteins in the WGCNA midnightblue module. (**B**) Nine candidate hub proteins identified through topological analysis of the PPI network. (**C**) AUC heatmap showing the performance of the eligible machine-learning strategies in the development cohort and two independent evaluation cohorts. GSE20685 was used as the development cohort, whereas GSE16446 and GSE48390 were used as evaluation cohorts. Models were ordered according to their mean AUC across GSE16446 and GSE48390; the development-cohort AUC was displayed but was not used for model ranking. The right-hand column represents the mean AUC across the two evaluation cohorts. (**D**,**E**) Global SHAP feature importance for the highest-ranked glmBoost–random forest model in GSE16446 (**D**) and GSE48390 (**E**). The seven highest-ranking features are displayed according to their mean absolute SHAP values. SLC27A2 ranked second in GSE16446 and first in GSE48390. (**F**) Pan-cancer comparison of SLC27A2 expression between tumour and normal tissues using data from The Cancer Genome Atlas (TCGA) and Genotype-Tissue Expression (GTEx) projects. Comparisons were performed using the Wilcoxon rank-sum test. (**G**) Unpaired comparison of SLC27A2 expression between breast cancer and normal breast tissues in the TCGA-BRCA cohort using the Wilcoxon rank-sum test. (**H**) Paired comparison of SLC27A2 expression between breast cancer tissues and matched adjacent normal tissues using the Wilcoxon signed-rank test. Expression levels are presented as log_2_(TPM + 1). ** *p* < 0.01; *** *p* < 0.001. AUC, area under the receiver operating characteristic curve; PPI, protein–protein interaction; SHAP, SHapley Additive exPlanations; TCGA, The Cancer Genome Atlas; TPM, transcripts per million; WGCNA, weighted gene co-expression network analysis.

To further prioritise candidate proteins, a PPI network was constructed for the 84 overlapping proteins using the STRING database and visualised with Cytoscape (Figure 2A). Topological analysis of the PPI network was subsequently performed. Nodes with above-average degree, closeness centrality, and clustering coefficient values were defined as core candidates, leading to the identification of nine proteins: ACSL1, ACSL3, RRAGA, DBT, SDR16C5, OSGEP, SLC27A2, HMGCL, and CRYL1 (Figure 2B).

We next evaluated the prognostic relevance of the nine candidate genes using three independent GEO breast cancer cohorts with available survival information. A total of 127 machine-learning strategies were developed using GSE20685 as the development cohort and GSE16446 and GSE48390 as evaluation cohorts. Models were ranked according to their mean AUC across the two evaluation cohorts. The glmBoost–random forest model achieved the highest mean evaluation AUC of 0.678 (Figure 2C). Its AUC was 0.635 (95% CI: 0.490–0.779) in GSE16446 and 0.721 (95% CI: 0.591–0.851) in GSE48390. These findings indicated moderate and cohort-dependent discriminative performance.

SHAP analysis was subsequently performed separately in the two evaluation cohorts to interpret the glmBoost–random forest model. Based on the mean absolute SHAP values, *SLC27A2* ranked second in GSE16446 and first in GSE48390 (Figure 2D,E). Although the relative importance of the other genes varied between cohorts, *SLC27A2* consistently remained among the two most influential features. This cross-cohort consistency supported the prioritisation of *SLC27A2* for subsequent functional and mechanistic investigation.

### 3.3. SLC27A2 Overexpression Promotes Malignant Phenotypes In Vitro

To further investigate the expression pattern of *SLC27A2*, we performed a pan-cancer analysis by integrating TCGA and GTEx datasets. The results revealed that *SLC27A2* is abnormally elevated in tumour tissues from various cancer types, including BC (Figure 2F). Consistent with the above findings, analysis of the TCGA-BRCA cohort alone confirmed that *SLC27A2* was significantly overexpressed in BC in both paired and unpaired comparisons (Figure 2G,H). These findings suggest that *SLC27A2* may play a potential role in BC.

**Figure 3:**
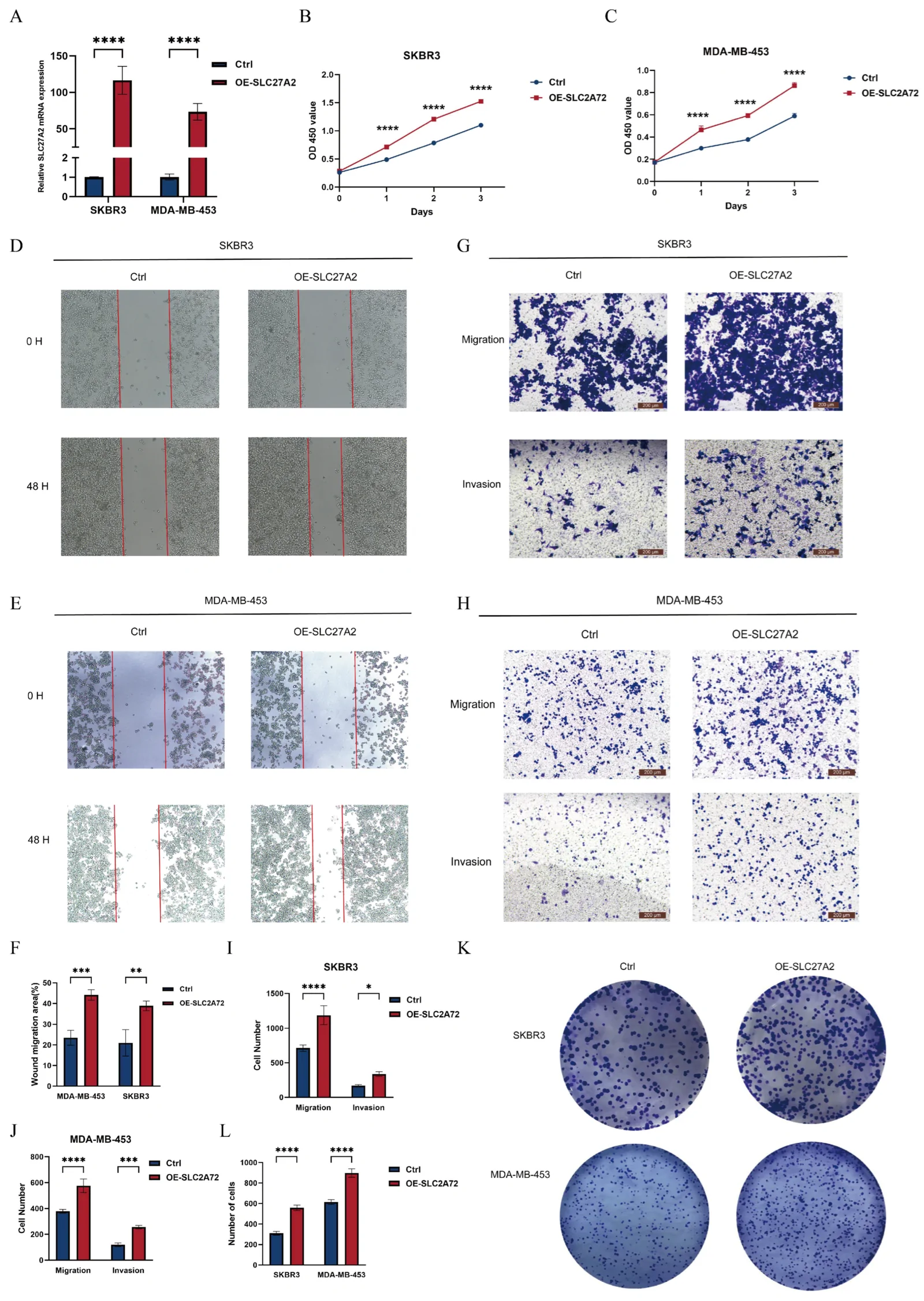
SLC27A2 overexpression promotes malignant phenotypes in HER2-positive breast cancer cells. (**A**) Validation of SLC27A2 overexpression efficiency in SKBR3 and MDA-MB-453 cells by qRT-PCR. Data were analysed using two-way ANOVA followed by Šídák’s multiple-comparisons test. (**B**,**C**) CCK-8 assays evaluating cell proliferation following SLC27A2 overexpression in SKBR3 (**B**) and MDA-MB-453 (**C**) cells. Data were analysed using two-way ANOVA followed by Šídák’s multiple-comparisons test. (**D**,**E**) Representative wound-healing images obtained at 0 and 48 h after scratching in SKBR3 (**D**) and MDA-MB-453 (**E**) cells. (**F**) Quantification of wound closure in both cell lines. Data were analysed using two-way ANOVA followed by Šídák’s multiple-comparisons test. (**G**,**H**) Representative Transwell migration and invasion images from SKBR3 (**G**) and MDA-MB-453 (**H**) cells. (**I**,**J**) Quantification of migrated and invaded cells in SKBR3 (**I**) and MDA-MB-453 (**J**) cells. Comparisons between the control and OE-SLC27A2 groups were performed using multiple two-tailed unpaired Student’s *t*-tests with Holm–Šídák correction. (**K**) Representative colony formation images following SLC27A2 overexpression in SKBR3 and MDA-MB-453 cells. (**L**) Quantification of colony numbers. Comparisons between the control and OE-SLC27A2 groups in each cell line were performed using multiple two-tailed unpaired Student’s *t*-tests with Holm–Šídák correction. Data are presented as the mean ± SD from three independent biological experiments. Technical replicates were averaged before statistical analysis. * *p* < 0.05; ** *p* < 0.01; *** *p* < 0.001; **** *p* < 0.0001. BC, breast cancer; CCK-8, Cell Counting Kit-8; Ctrl, control; OE-SLC27A2, SLC27A2 overexpression; qRT-PCR, quantitative real-time polymerase chain reaction; SD, standard deviation.

To further analyse the role of *SLC27A2* in BC and its influence on sensitivity to pyrotinib, we conducted in vitro studies using two HER2-positive BC cell lines (SKBR3 and MDA-MB-453). We first used a plasmid-encoding *SLC27A2* to overexpress *SLC27A2* in BC cells. qRT-PCR experiments confirmed that *SLC27A2* had been successfully overexpressed (Figure 3A). We then performed a series of functional assays to investigate the biological effects of *SLC27A2* overexpression in BC cells. CCK-8 assays showed that *SLC27A2* overexpression significantly enhanced cell proliferation (Figure 3B,C). Wound-healing assays showed increased wound closure following *SLC27A2* overexpression. Consistently, Transwell assays demonstrated increased numbers of migrated and invaded cells in both SKBR3 and MDA-MB-453 cells (Figure 3D–J). The concordant findings obtained using two complementary assay approaches support an association between *SLC27A2* overexpression and enhanced migratory and invasive phenotypes. In addition, colony formation assays demonstrated that overexpression of *SLC27A2* significantly enhanced the clonogenic capacity of BC cells in vitro (Figure 3K,L). Collectively, these findings indicate that *SLC27A2* overexpression promotes malignant cellular phenotypes in vitro.

### 3.4. SLC27A2 Knockdown Suppresses Malignant Phenotypes In Vitro

**Figure 4:**
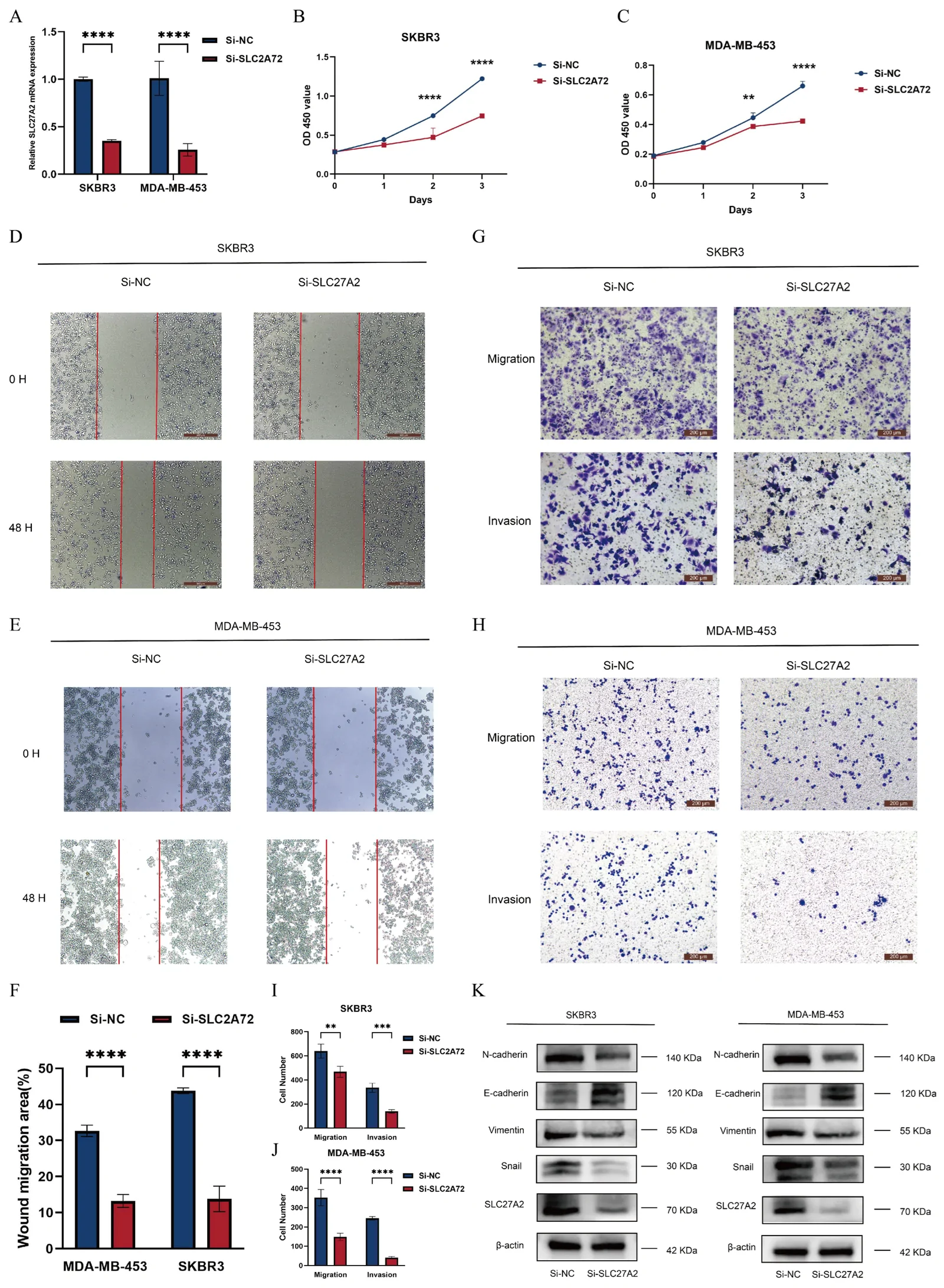
SLC27A2 knockdown suppresses malignant phenotypes in HER2-positive breast cancer cells. (**A**) Validation of SLC27A2 knockdown efficiency in SKBR3 and MDA-MB-453 cells by qRT-PCR. Comparisons between the si-NC and si-SLC27A2 groups in each cell line were performed using multiple two-tailed unpaired Student’s *t*-tests with Holm–Šídák correction. (**B**,**C**) CCK-8 assays evaluating cell proliferation following SLC27A2 knockdown in SKBR3 (**B**) and MDA-MB-453 (**C**) cells. Data were analysed using two-way ANOVA followed by Šídák’s multiple-comparisons test. (**D**,**E**) Representative wound-healing images obtained at 0 and 48 h after scratching in SKBR3 (**D**) and MDA-MB-453 (**E**) cells. (**F**) Quantification of wound closure. Comparisons between the si-NC and si-SLC27A2 groups in each cell line were performed using multiple two-tailed unpaired Student’s *t*-tests with Holm–Šídák correction. (**G**,**H**) Representative Transwell migration and invasion images from SKBR3 (**G**) and MDA-MB-453 (**H**) cells. (**I**,**J**) Quantification of migrated and invaded cells in SKBR3 (**I**) and MDA-MB-453 (**J**) cells. Comparisons between the si-NC and si-SLC27A2 groups were performed using multiple two-tailed unpaired Student’s *t*-tests with Holm–Šídák correction. (**K**) Representative Western blot images showing the expression of the epithelial–mesenchymal transition markers E-cadherin, N-cadherin, Vimentin, and Snail, together with SLC27A2, following SLC27A2 knockdown in SKBR3 and MDA-MB-453 cells. β-actin was used as the loading control, and 30 μg of total protein was loaded per lane. Data are presented as the mean ± SD from three independent biological experiments. Technical replicates were averaged before statistical analysis. ** *p* < 0.01; *** *p* < 0.001; **** *p* < 0.0001. BC, breast cancer; CCK-8, Cell Counting Kit-8; EMT, epithelial–mesenchymal transition; qRT-PCR, quantitative real-time polymerase chain reaction; SD, standard deviation; si-NC, non-targeting control small interfering RNA. Original western blots are presented in Supplementary Materials.

We next investigated the effects of *SLC27A2* knockdown on malignant cellular phenotypes in vitro. siRNA was used to knock down *SLC27A2* expression (Figure 4A). CCK-8 assays showed that *SLC27A2* knockdown significantly inhibited cell proliferation (Figure 4B,C). In addition, *SLC27A2* knockdown reduced wound closure in both cell lines. Consistently, Transwell migration and invasion assays showed reduced numbers of migrated and invaded cells following *SLC27A2* knockdown (Figure 4D–J). These concordant findings support an association between *SLC27A2* knockdown and the suppression of migratory and invasive phenotypes. Given that *SLC27A2* knockdown markedly inhibited the migratory and invasive abilities of BC cells, we next investigated whether these effects were associated with epithelial–mesenchymal transition (EMT). Western blot analysis demonstrated that SLC27A2 knockdown significantly inhibited EMT, as evidenced by the increased expression of the epithelial marker E-cadherin and reduced expression of mesenchymal markers, including N-cadherin, Snail and Vimentin (Figure 4K). Collectively, these results indicate that *SLC27A2* knockdown suppresses BC progression in vitro.

### 3.5. SLC27A2 Knockdown Increases Pyrotinib Sensitivity In Vitro and Interacts with Pyrotinib Treatment in Reducing Endpoint Xenograft Weight

**Figure 5:**
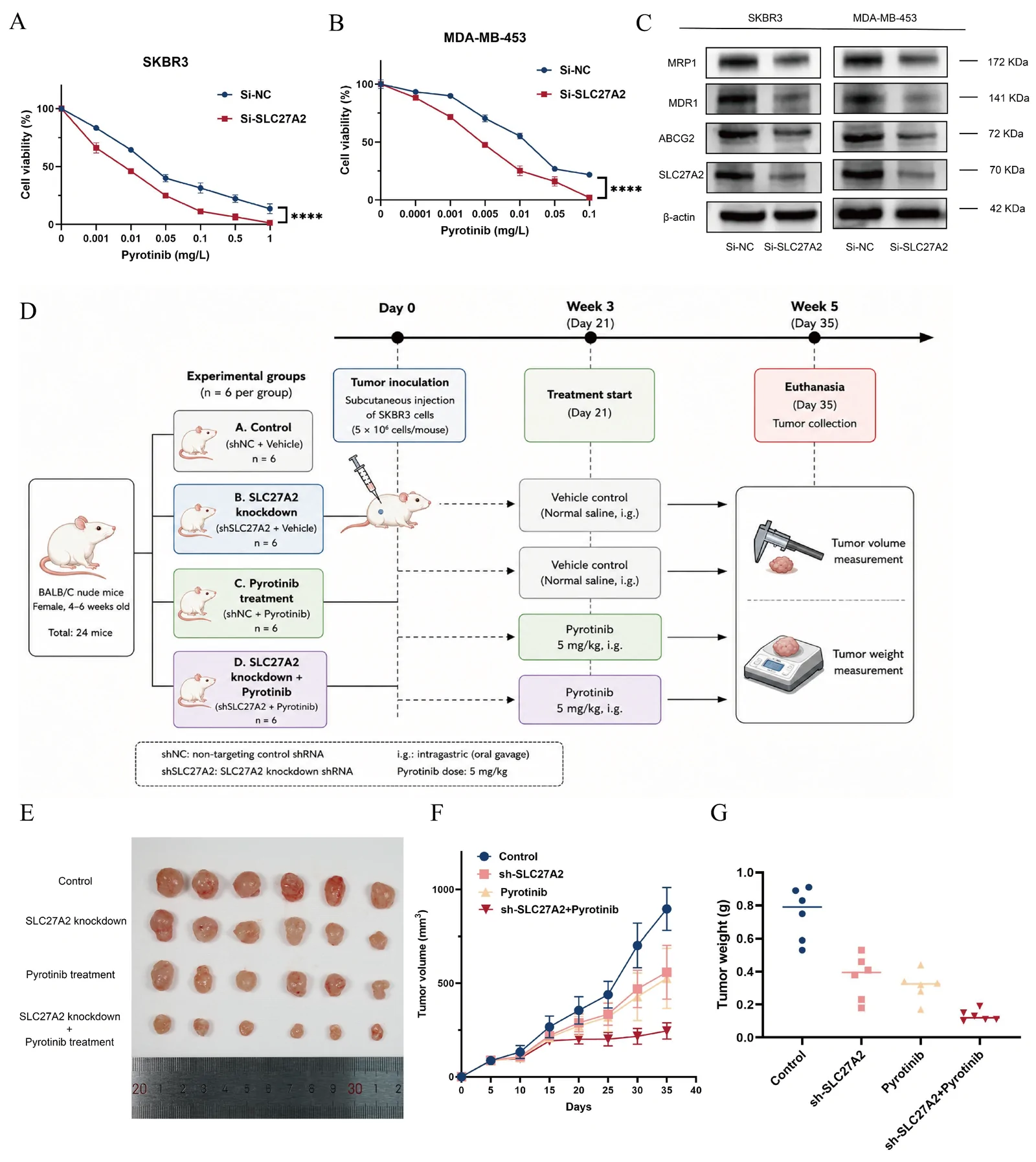
SLC27A2 knockdown increases pyrotinib sensitivity in vitro and shows an interaction with pyrotinib treatment for endpoint xenograft weight. (**A**,**B**) Pyrotinib dose–response curves in control and SLC27A2-knockdown SKBR3 (**A**) and MDA-MB-453 (**B**) cells. Cell viability was measured after 48 h of pyrotinib treatment. Data are presented as the mean ± SD from three independent biological experiments and were analysed using two-way ANOVA; **** *p* < 0.0001 for the overall effect of SLC27A2 knockdown. (**C**) Representative Western blot images showing the expression of MRP1, MDR1, ABCG2, and SLC27A2 following SLC27A2 knockdown in SKBR3 and MDA-MB-453 cells. β-actin was used as the loading control, and 30 μg of total protein was loaded per lane. (**D**) Schematic representation of the xenograft experiment. Twenty-four mice were randomly allocated to four groups in a 2 × 2 factorial design, with six mice per group: shNC plus vehicle, shSLC27A2 plus vehicle, shNC plus pyrotinib, and shSLC27A2 plus pyrotinib. Tumour measurements were performed by an investigator blinded to group allocation. (**E**) Images of excised xenograft tumours from the four experimental groups. (**F**) Longitudinal tumour-volume curves. Post-treatment tumour volumes from day 20 to day 35 were analysed using a linear mixed-effects model, with mouse identity included as a random effect. SLC27A2 knockdown, pyrotinib treatment, time, and their interaction terms were included as fixed effects. Significant knockdown-by-time and pyrotinib-by-time effects were observed (both *p* < 0.0001), whereas the knockdown-by-pyrotinib-by-time interaction was not statistically significant (*p* = 0.266). Data are presented as the mean ± SD; *n* = 6 mice per group. (**G**) Endpoint tumour weights. Individual data points and group means are shown. Tumour weights were analysed on the original measurement scale using two-way ANOVA, with SLC27A2 knockdown and pyrotinib treatment included as the two experimental factors. Significant main effects of SLC27A2 knockdown and pyrotinib treatment were observed (both *p* < 0.0001), together with a significant interaction between the two factors (*p* for interaction = 0.041); *n* = 6 mice per group. All statistical tests were two-sided. ABCG2, ATP-binding cassette subfamily G member 2; BC, breast cancer; MDR1, multidrug resistance protein 1; MRP1, multidrug resistance-associated protein 1; SD, standard deviation. Original western blots are presented in Supplementary Materials.

We next investigated the association between SLC27A2 and pyrotinib sensitivity. CCK-8 assays showed that *SLC27A2* knockdown significantly increased the inhibitory effect of pyrotinib on cell viability in SKBR3 and MDA-MB-453 cells, supporting increased pyrotinib sensitivity in vitro (Figure 5A,B). Western blot analysis further showed that SLC27A2 knockdown reduced the expression of the multidrug resistance-associated proteins MRP1, MDR1, and ABCG2 (Figure 5C). These findings suggest that the suppression of drug resistance-associated proteins may contribute to increased pyrotinib sensitivity following SLC27A2 knockdown.

We subsequently used a xenograft model with a 2 × 2 factorial design to evaluate the effects of SLC27A2 knockdown and pyrotinib treatment in vivo (Figure 5D). Both SLC27A2 knockdown and pyrotinib treatment significantly reduced tumour growth, and the shSLC27A2 plus pyrotinib group exhibited the lowest tumour volume and weight among the four groups (Figure 5E–G). Two-way ANOVA of endpoint tumour weight on the original measurement scale identified a statistically significant interaction between SLC27A2 knockdown and pyrotinib treatment (*p* for interaction = 0.041), indicating that the effect of pyrotinib on endpoint tumour weight differed according to SLC27A2 knockdown status. However, the corresponding interaction was not statistically significant for longitudinal tumour growth (*p* = 0.266) or tumour volume on day 35 (*p* = 0.583). These findings demonstrate a pronounced inhibitory effect of SLC27A2 knockdown on xenograft growth and suggest an interaction with pyrotinib treatment for endpoint tumour weight, although this interaction was not consistently detected across all tumour-growth outcomes. No animals died or reached the prespecified humane endpoints, and body weight remained generally stable across all groups throughout the experimental period (Supplementary Table S3).

### 3.6. SLC27A2 Is Associated with Pyrotinib Sensitivity and PPARα-Related Fatty Acid Metabolism

**Figure 6:**
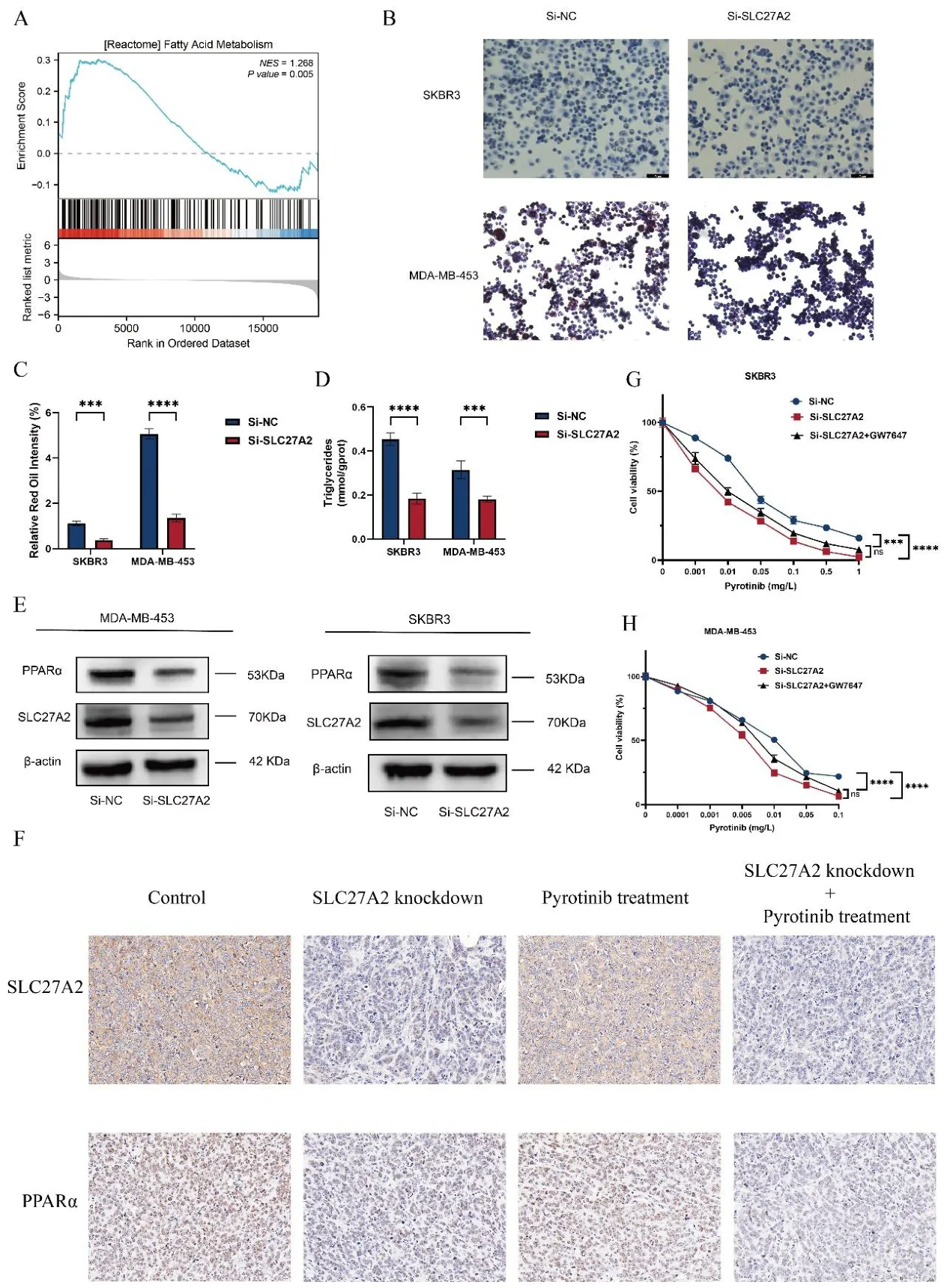
SLC27A2 is associated with pyrotinib sensitivity and PPARα-related fatty acid metabolism. (**A**) Gene set enrichment analysis showing enrichment of the Reactome fatty acid metabolism pathway according to SLC27A2 expression in the TCGA-BRCA cohort. (**B**) Representative Oil Red O staining images showing intracellular lipid accumulation in control and SLC27A2-knockdown SKBR3 and MDA-MB-453 cells. (**C**) Quantification of Oil Red O staining intensity. Comparisons between the si-NC and si-SLC27A2 groups in each cell line were performed using multiple two-tailed unpaired Student’s *t*-tests with Holm–Šídák correction. (**D**) Intracellular triglyceride levels in control and SLC27A2-knockdown SKBR3 and MDA-MB-453 cells. Comparisons between the si-NC and si-SLC27A2 groups in each cell line were performed using multiple two-tailed unpaired Student’s *t*-tests with Holm–Šídák correction. (**E**) Representative Western blot images showing PPARα and SLC27A2 expression following SLC27A2 knockdown in SKBR3 and MDA-MB-453 cells. β-actin was used as the loading control, and 30 μg of total protein was loaded per lane. (**F**) Representative immunohistochemical staining of SLC27A2 and PPARα in xenograft tumours from the control, SLC27A2-knockdown, pyrotinib treatment, and SLC27A2-knockdown plus pyrotinib treatment groups. (**G**,**H**) Pyrotinib dose–response curves showing the effects of pharmacological PPARα activation with GW7647 in SLC27A2-knockdown SKBR3 (**G**) and MDA-MB-453 (**H**) cells. Data were analysed using two-way ANOVA followed by Šídák’s multiple-comparisons test. Data are presented as the mean ± SD from three independent biological experiments. Technical replicates were averaged before statistical analysis. *** *p* < 0.001; **** *p* < 0.0001; ns, not significant. GSEA, gene set enrichment analysis; IHC, immunohistochemistry; NES, normalised enrichment score; PPARα, peroxisome proliferator-activated receptor alpha; SD, standard deviation; si-NC, non-targeting control small interfering RNA; TCGA-BRCA, The Cancer Genome Atlas Breast Invasive Carcinoma cohort. Original western blots are presented in Supplementary Materials.

To explore the potential mechanisms underlying the association between SLC27A2 and pyrotinib sensitivity, we first performed gene set enrichment analysis using the TCGA-BRCA cohort. Genes associated with *SLC27A2* expression were enriched in the Reactome fatty acid metabolism pathway, suggesting a potential relationship between *SLC27A2* and lipid metabolic activity in BC (Figure 6A). Oil Red O staining subsequently showed that *SLC27A2* knockdown reduced intracellular lipid accumulation in SKBR3 and MDA-MB-453 cells (Figure 6B,C). Consistently, intracellular triglyceride levels were significantly decreased following *SLC27A2* knockdown (Figure 6D).

Given the central role of PPARα in fatty acid metabolism [12,13], we next examined its protein expression. Western blot analysis showed that SLC27A2 knockdown was accompanied by reduced PPARα protein expression in both cell lines (Figure 6E). IHC staining of xenograft tissues further showed the sustained suppression of SLC27A2 in tumours derived from SLC27A2-knockdown cells, accompanied by weaker PPARα staining (Figure 6F).

To further investigate whether PPARα activity contributes to the effect of SLC27A2 knockdown on pyrotinib response, *SLC27A2*-knockdown cells were treated with the selective PPARα agonist GW7647. Pharmacological activation of PPARα partially attenuated the increased pyrotinib sensitivity induced by *SLC27A2* knockdown in both cell lines (Figure 6G,H). Collectively, these findings support an association among SLC27A2 expression, PPARα-related fatty acid metabolism, and pyrotinib response. However, they do not establish a direct regulatory relationship between SLC27A2 and PPARα.

### 3.7. Clinical Association Between SLC27A2 Expression and Response to Pyrotinib-Containing Neoadjuvant Therapy

To evaluate the clinical association between pre-treatment SLC27A2 expression and pathological response, we analysed an independent, non-overlapping retrospective cohort of 103 patients with HER2-positive BC who received pyrotinib-containing neoadjuvant therapy at the same institution. Among them, 65 patients achieved pCR, whereas 38 had residual disease (non-pCR). IHC was performed on BC tissues collected before treatment, revealing 37 cases with high SLC27A2 expression and 66 cases with low SLC27A2 expression.

Clinicopathological analysis showed that pathological response was significantly associated with ER status, PR status, Ki-67 expression, and SLC27A2 expression (Table 1). Consistently, univariate logistic regression identified ER status, PR status, Ki-67 expression, and SLC27A2 expression as significant predictors of pCR. Multivariate analysis further demonstrated that only Ki-67 expression and SLC27A2 expression remained independent predictors of pathological response (Table 2). Notably, patients with high SLC27A2 expression were significantly less likely to achieve pCR following pyrotinib-containing neoadjuvant therapy than those with low SLC27A2 expression (OR = 0.10, 95% CI: 0.03–0.31, *p* < 0.001), indicating that elevated SLC27A2 expression is independently associated with a poorer response to pyrotinib-containing therapy. These clinical findings further support our experimental results, suggesting that SLC27A2 is a promising predictive biomarker for response to pyrotinib-containing neoadjuvant therapy in BC.

**Table 1:** Association between clinicopathological characteristics and pathological response in patients receiving pyrotinib-containing neoadjuvant therapy.

Characteristics	Total (*N* = 103)	NAC Efficacy		*p* Value
		Non-pCR (*N* = 38)	pCR (*N* = 65)	
**Age**				0.218
<60	80 (77.67)	27 (71.05)	53 (81.54)	
≥60	23 (22.33)	11 (28.95)	12 (18.46)	
**Menstrual status**				0.659
Postmenopausal	49 (47.57)	17 (44.74)	32 (49.23)	
Premenopausal	54 (52.43)	21 (55.26)	33 (50.77)	
**T stage**				0.888
T1	12 (11.65)	4 (10.53)	8 (12.31)	
T2	72 (69.90)	26 (68.42)	46 (70.77)	
T3	8 (7.77)	4 (10.53)	4 (6.15)	
T4	11 (10.68)	4 (10.53)	7 (10.77)	
**N stage**				0.245
N0	10 (9.71)	1 (2.63)	9 (13.85)	
N1	69 (66.99)	29 (76.32)	40 (61.54)	
N2	8 (7.77)	3 (7.89)	5 (7.69)	
N3	16 (15.53)	5 (13.16)	11 (16.92)	
**AJCC stage**				0.774
I	2 (1.94)	0 (0.00)	2 (3.08)	
II	65 (63.11)	24 (63.16)	41 (63.08)	
III	36 (34.95)	14 (36.84)	22 (33.85)	
**ER status**				<0.001
Positive	62 (60.19)	31 (81.58)	31 (47.69)	
Negative	41 (39.81)	7 (18.42)	34 (52.31)	
**PR status**				<0.001
Positive	51 (49.51)	28 (73.68)	23 (35.38)	
Negative	52 (50.49)	10 (26.32)	42 (64.62)	
**K i67**				0.005
<30%	27 (26.21)	16 (42.11)	11 (16.92)	
≥30%	76 (73.79)	22 (57.89)	54 (83.08)	
**SLC27A2 expression**				<0.001
Low	66 (64.08)	14 (36.84)	52 (80.00)	
High	37 (35.92)	24 (63.16)	13 (20.00)	
**TILS, M (Q_1_, Q_3_)**	0.08(0.05, 0.15)	0.05 (0.05, 0.08)	0.08 (0.05, 0.15)	0.056

**Table 2:** Univariate and multivariate logistic regression analyses of factors associated with pathological complete response.

Characteristics	Univariate Analysis					Multivariate Analysis				
	β	S.E	Z	*p*	OR (95%CI)	β	S.E	Z	*p*	OR (95%CI)
**Age**										
<60					1.00 (Reference)					
≥60	−0.59	0.48	−1.22	0.221	0.56 (0.22~1.42)					
**T stage**										
T1					1.00 (Reference)					
T2	−0.12	0.66	−0.19	0.853	0.88 (0.24~3.22)					
T3	−0.69	0.94	−0.74	0.459	0.50 (0.08~3.13)					
T4	−0.13	0.88	−0.15	0.879	0.88 (0.16~4.87)					
**N stage**										
N0					1.00 (Reference)					
N1	−1.88	1.08	−1.73	0.083	0.15 (0.02~1.28)					
N2	−1.69	1.28	−1.32	0.188	0.19 (0.02~2.29)					
N3	−1.41	1.18	−1.19	0.234	0.24 (0.02~2.49)					
**Menstrual status**										
Premenopausal					1.00 (Reference)					
Postmenopausal	0.18	0.41	0.44	0.660	1.20 (0.54~2.67)					
**AJCC stage**										
I					1.00 (Reference)					
II	−15.03	1029.12	−0.01	0.988	0.00 (0.00~Inf)					
III	−15.11	1029.12	−0.01	0.988	0.00 (0.00~Inf)					
**ER status**										
Positive					1.00 (Reference)					1.00 (Reference)
Negative	1.58	0.49	3.25	0.001	4.86 (1.87~12.61)	1.06	0.65	1.63	0.103	2.89 (0.81~10.38)
**PR status**										
Positive					1.00 (Reference)					1.00 (Reference)
Negative	1.63	0.45	3.62	<0.001	5.11 (2.11~12.37)	1.17	0.61	1.91	0.056	3.22 (0.97~10.71)
**Ki67**										
<30%					1.00 (Reference)					1.00 (Reference)
≥30%	1.27	0.47	2.73	0.006	3.57 (1.43~8.90)	1.56	0.59	2.63	0.009	4.75 (1.49~15.20)
**SLC27A2**										
Low SLC27A2					1.00 (Reference)					1.00 (Reference)
High SLC27A2	−1.93	0.46	−4.21	<0.001	0.15 (0.06~0.36)	−2.28	0.56	−4.06	<0.001	0.10 (0.03~0.31)

## 4. Discussion

Pyrotinib is one of the most widely used targeted therapies for the clinical treatment of HER2-positive BC and has demonstrated remarkable efficacy [14]. However, intrinsic and acquired resistance represent major challenges that limit its long-term therapeutic benefits [15]. Although several mechanisms underlying resistance to HER2-targeted therapies have been proposed, reliable biomarkers for predicting pyrotinib response and the molecular basis of resistance remain poorly understood. In this study, we integrated proteomic profiling, network analysis, machine learning, SHAP interpretation, functional experiments, and retrospective clinical validation to investigate candidate factors associated with pyrotinib response and BC prognosis. Among 127 machine-learning strategies, the glmBoost–random forest model achieved the highest mean AUC across two independent evaluation cohorts. SHAP analysis further showed that *SLC27A2* consistently ranked among the two most influential features in both cohorts, supporting its prioritisation for subsequent investigation. Functional experiments demonstrated that *SLC27A2* promoted malignant phenotypes in HER2-positive BC cells and that its inhibition enhanced the anti-tumour effects of pyrotinib. Mechanistically, our findings suggested that PPARα-related fatty acid metabolism may contribute to the association between *SLC27A2* expression and pyrotinib sensitivity. The retrospective clinical analysis further showed that high-pre-treatment SLC27A2 expression was independently associated with a lower likelihood of achieving pCR after pyrotinib-containing neoadjuvant therapy. Collectively, these findings support SLC27A2 as a candidate response-associated biomarker and potential therapeutic target warranting further investigation.

SLC27A2, also known as fatty acid transport protein 2 (FATP2), is a member of the FATP family, playing a central role in the uptake and activation of long-chain fatty acids [16,17]. Emerging evidence suggests SLC27A2 has an important role in tumour progression [18]. For example, in anaplastic thyroid cancer, SLC27A2 overexpression promotes tumour cell proliferation and migration [19]. In ovarian cancer, SLC27A2 has been implicated in chemoresistance via the regulation of miR-411 [20]. Additionally, high SLC27A2 expression is associated with suppressed anti-tumour immunity and poor prognosis in acute lymphoblastic leukaemia [21]. These findings suggest that *SLC27A2* acts as an oncogene across multiple cancer types. To our knowledge, this study provides initial evidence that SLC27A2 promotes malignant phenotypes in HER2-positive BC and is associated with reduced pyrotinib sensitivity, thereby extending its potential oncogenic role and supporting its further investigation as a therapeutic target.

Mechanistically, our findings suggest that alterations in fatty acid metabolism may contribute to the association between SLC27A2 expression and pyrotinib sensitivity. Tumour growth is highly dependent on the availability of fatty acids [22]. In 2011, Hanahan and Weinberg identified metabolic reprogramming, including fatty acid metabolism, as a hallmark of cancer [23,24]. Subsequent studies have increasingly shown that fatty acid metabolic reprogramming is also a key driver of drug resistance in cancer therapy [25,26,27]. By enhancing fatty acid uptake and utilisation, tumour cells can sustain energy production, membrane biosynthesis, and metabolic homeostasis under therapeutic stress, promoting survival and reducing drug sensitivity [28]. As a key fatty acid transporter, SLC27A2 plays a critical role in this process. By facilitating the uptake and activation of long-chain fatty acids [29], SLC27A2 may contribute to fatty acid metabolic reprogramming, potentially creating a metabolic environment that supports tumour progression and reduced treatment sensitivity. Supporting this hypothesis, GSEA showed that *SLC27A2* is closely associated with fatty acid metabolism. Furthermore, *SLC27A2* knockdown significantly reduced lipid droplet accumulation and intracellular triglyceride levels, highlighting its role in lipid metabolic remodelling in HER2-positive BC.

Our findings suggest that PPARα-related metabolic activity may contribute to the association between SLC27A2 and pyrotinib sensitivity. PPARα is a ligand-activated nuclear receptor that plays a central role in fatty acid transport, activation, and β-oxidation [12,13]. Long-chain fatty acids and their derivatives can serve as endogenous PPARα ligands and regulate downstream metabolic programmes involved in fatty acid oxidation, mitochondrial function, and energy homeostasis [30]. In the present study, SLC27A2 knockdown reduced PPARα protein expression in HER2-positive BC cells. Consistent changes in PPARα staining were also observed in xenograft tumours derived from SLC27A2-knockdown cells. Moreover, pharmacological activation of PPARα with GW7647 partially attenuated the increase in pyrotinib sensitivity induced by SLC27A2 knockdown. These results provide preliminary functional evidence supporting the involvement of PPARα-related fatty acid metabolism. Nevertheless, because the rescue experiment relied on pharmacological activation alone, the present findings do not establish a direct regulatory relationship between SLC27A2 and PPARα.

Beyond elucidating the molecular mechanism underlying pyrotinib resistance, our study further explored the clinical relevance of SLC27A2 as a predictive biomarker. Unlike previous studies that mainly focused on the biological functions and regulatory mechanisms of resistance-related molecules, we additionally performed a retrospective clinical analysis using tumour samples from patients receiving pyrotinib-containing neoadjuvant therapy. The results demonstrated that SLC27A2 expression was significantly associated with pathological response to pyrotinib-based treatment, and high SLC27A2 expression independently predicted a lower probability of achieving pCR. These findings suggest that SLC27A2 is not only a potential therapeutic target for overcoming pyrotinib resistance but also a promising biomarker for identifying patients who are more likely to benefit from pyrotinib-based therapy.

From a translational perspective, targeting SLC27A2 may represent a potential strategy for improving pyrotinib sensitivity, particularly in HER2-positive BC with high SLC27A2 expression. However, the efficacy and safety of combining pharmacological SLC27A2 inhibition with pyrotinib require further evaluation in clinically relevant preclinical models.

This study has several limitations. First, the exploratory proteomic analysis was based on a small, selectively sampled discovery cohort, which may have introduced sampling bias. Moreover, the retrospective clinical cohort was derived from a single institution, and external validation is currently lacking. Second, because the public cohorts used for machine-learning analysis lacked pyrotinib treatment information, the resulting model primarily reflects the prognostic relevance of the candidate genes and cannot directly establish their predictive value for pyrotinib response. Third, the mechanistic evidence linking SLC27A2 to PPARα-related fatty acid metabolism remains preliminary, and a direct regulatory relationship has not been established. Finally, although the factorial xenograft experiment supported an interaction between SLC27A2 knockdown and pyrotinib for endpoint tumour weight, further studies using pharmacological inhibitors, patient-derived models, and larger animal cohorts are required before clinical translation.

## 5. Conclusions

This study identifies SLC27A2 as a candidate factor associated with BC prognosis and reduced pyrotinib sensitivity in HER2-positive BC. SLC27A2 knockdown suppressed malignant cellular phenotypes and increased pyrotinib sensitivity in vitro. In vivo, SLC27A2 knockdown markedly reduced xenograft growth and showed a significant interaction with pyrotinib treatment for endpoint tumour weight. The observed changes in lipid accumulation and PPARα expression, together with the pharmacological rescue results, suggest that PPARα-related fatty acid metabolism may contribute to this association. In the retrospective clinical cohort, high-pre-treatment SLC27A2 expression was independently associated with a lower likelihood of achieving pCR following pyrotinib-containing neoadjuvant therapy. These findings support SLC27A2 as a potential response-associated biomarker and therapeutic target, although external clinical validation and further mechanistic investigation are required.

## Data Availability

Detailed analytical procedures, machine-learning settings, model-performance results, and numerical data supporting the principal findings are provided in Methods and Supplementary Materials. Individual-level clinical data are not publicly available because they contain potentially sensitive patient information and are subject to institutional ethical and privacy restrictions. De-identified clinical data may be made available from the corresponding author upon reasonable request and following approval by the institutional ethics committee.

## Supplementary Materials

The following supporting information can be downloaded at https://www.mdpi.com/article/10.3390/cancers18162702/s1, **Table S1.** Detailed configurations and performance of the 127 machine-learning strategies. **Table S2.** Detailed information on the primary and secondary antibodies used for Western blotting and immunohistochemistry. **Table S3.** Body-weight measurements of mice in the four experimental groups during the xenograft experiment. Uncropped gels for Figure 4K. Uncropped gels for Figure 5C. Uncropped gels for Figure 6E.

## References

[1] Bray F., Laversanne M., Sung H., Ferlay J., Siegel R.L., Soerjomataram I., Jemal A. (2024). Global cancer statistics 2022: GLOBOCAN estimates of incidence and mortality worldwide for 36 cancers in 185 countries. CA Cancer J. Clin..

[2] Qi X., Shi Q., Xuhong J., Zhang Y., Jiang J. (2023). Pyrotinib-based therapeutic approaches for HER2-positive breast cancer: The time is now. Breast Cancer Res. BCR.

[3] Liu X., Zhang X., Shao Z., Zhong X., Ding X., Wu L., Chen J., He P., Cheng Y., Zhu K. (2023). Pyrotinib and chrysin synergistically potentiate autophagy in HER2-positive breast cancer. Signal Transduct. Target. Ther..

[4] Loibl S., Gianni L. (2017). HER2-positive breast cancer. Lancet.

[5] (2021). Early Breast Cancer Trialists’ Collaborative group (EBCTCG). Trastuzumab for early-stage, HER2-positive breast cancer: A meta-analysis of 13 864 women in seven randomised trials. Lancet Oncol..

[6] Ma F., Yan M., Li W., Ouyang Q., Tong Z., Teng Y., Wang Y., Wang S., Geng C., Luo T. (2023). Pyrotinib versus placebo in combination with trastuzumab and docetaxel as first line treatment in patients with HER2 positive metastatic breast cancer (PHILA): Randomised, double blind, multicentre, phase 3 trial. BMJ.

[7] Werter I.M., Remmelzwaal S., Burchell G.L., de Gruijl T.D., Konings I.R., van der Vliet H.J., Menke-van der Houven van Oordt C.W. (2022). Systemic Therapy for Patients with HER2-Positive Breast Cancer and Brain Metastases: A Systematic Review and Meta-Analysis. Cancers.

[8] Wu J., Jiang Z., Liu Z., Yang B., Yang H., Tang J., Wang K., Liu Y., Wang H., Fu P. (2022). Neoadjuvant pyrotinib, trastuzumab, and docetaxel for HER2-positive breast cancer (PHEDRA): A double-blind, randomized phase 3 trial. BMC Med..

[9] Zhang Y., Yang C., Jia Y., Luo R., Wang J., Han M., Liu Y., Wang X., Shi L., Yang J. (2026). Efficacy and safety of pyrotinib combined with trastuzumab and chemotherapy in patients with HER2-positive locally advanced breast cancer: A multicenter real-world study. Am. J. Transl. Res..

[10] Giuliano A.E., Edge S.B., Hortobagyi G.N. (2018). Eighth Edition of the AJCC Cancer Staging Manual: Breast Cancer. Ann. Surg. Oncol..

[11] Sha R., Xu Y., Yuan C., Sheng X., Wu Z., Peng J., Wang Y., Lin Y., Zhou L., Xu S. (2021). Predictive and prognostic impact of ferroptosis-related genes ACSL4 and GPX4 on breast cancer treated with neoadjuvant chemotherapy. EBioMedicine.

[12] Pawlak M., Lefebvre P., Staels B. (2015). Molecular mechanism of PPARα action and its impact on lipid metabolism, inflammation and fibrosis in non-alcoholic fatty liver disease. J. Hepatol..

[13] Gao J., Yuan S., Jin J., Shi J., Hou Y. (2015). PPARα regulates tumor progression, foe or friend?. Eur. J. Pharmacol..

[14] Ma X., Li Y., Li L., Gao C., Liu D., Li H., Zhao Z., Zhao B. (2022). Pyrotinib-based treatments in HER2-positive breast cancer patients with brain metastases. Ann. Med..

[15] Yang C., Xu Y., Lin Z., Zhang A., Li L., Ye Z., Zhang Q., Hu H., Ren G., Cheng P. (2025). Spatial discovery of pyrotinib overcoming HER2-positive breast cancer resistance by breaking fibroblast-induced immune barriers. Drug Resist. Updat. Rev. Comment. Antimicrob. Anticancer Chemother..

[16] Wang Y., Zhang Y., Zhang B., Khan F.I. (2026). The FATP2 axis in cancer: Structural informatics and implications for drug discovery. Drug Discov. Today.

[17] Park J., Jang J.Y., Kim J.H., Yi S.E., Lee Y.J., Yu M.S., Chung Y.S., Jang Y.J., Kim J.H., Kang K. (2025). SLC27A2 marks lipid peroxidation in nasal epithelial cells driven by type 2 inflammation in chronic rhinosinusitis with nasal polyps. Exp. Mol. Med..

[18] Veglia F., Tyurin V.A., Blasi M., De Leo A., Kossenkov A.V., Donthireddy L., To T.K.J., Schug Z., Basu S., Wang F. (2019). Fatty acid transport protein 2 reprograms neutrophils in cancer. Nature.

[19] Feng K., Ma R., Li H., Yin K., Du G., Chen X., Liu Z., Yin D. (2022). Upregulated SLC27A2/FATP2 in differentiated thyroid carcinoma promotes tumor proliferation and migration. J. Clin. Lab. Anal..

[20] Chen F.D., Chen H.H., Ke S.C., Zheng L.R., Zheng X.Y. (2018). SLC27A2 regulates miR-411 to affect chemo-resistance in ovarian cancer. Neoplasma.

[21] Lu L., Li J., Zheng Y., Luo L., Huang Y., Hu J., Chen Y. (2024). High expression of SLC27A2 predicts unfavorable prognosis and promotes inhibitory immune infiltration in acute lymphoblastic leukemia. Transl. Oncol..

[22] Koundouros N., Poulogiannis G. (2020). Reprogramming of fatty acid metabolism in cancer. Br. J. Cancer.

[23] Hanahan D., Weinberg R.A. (2011). Hallmarks of cancer: The next generation. Cell.

[24] Nong S., Han X., Xiang Y., Qian Y., Wei Y., Zhang T., Tian K., Shen K., Yang J., Ma X. (2023). Metabolic reprogramming in cancer: Mechanisms and therapeutics. MedComm.

[25] Tan Y., Li J., Zhao G., Huang K.C., Cardenas H., Wang Y., Matei D., Cheng J.X. (2022). Metabolic reprogramming from glycolysis to fatty acid uptake and beta-oxidation in platinum-resistant cancer cells. Nat. Commun..

[26] Nandi I., Ji L., Smith H.W., Avizonis D., Papavasiliou V., Lavoie C., Pacis A., Attalla S., Sanguin-Gendreau V., Muller W.J. (2024). Targeting fatty acid oxidation enhances response to HER2-targeted therapy. Nat. Commun..

[27] Chen M., Li W., Tao Y., Hu C., Ge R., Kang S., Yue P., Man C.H., Wang L., Yan X. (2026). SLC25A1 reprograms mitochondrial and fatty acid metabolism to promote the progression of acute myeloid leukemia. Haematologica.

[28] Vishwa R., BharathwajChetty B., Girisa S., Aswani B.S., Alqahtani M.S., Abbas M., Hegde M., Kunnumakkara A.B. (2024). Lipid metabolism and its implications in tumor cell plasticity and drug resistance: What we learned thus far?. Cancer Metastasis Rev..

[29] Tao L., Mohammad M.A., Milazzo G., Moreno-Smith M., Patel T.D., Zorman B., Badachhape A., Hernandez B.E., Wolf A.B., Zeng Z. (2022). MYCN-driven fatty acid uptake is a metabolic vulnerability in neuroblastoma. Nat. Commun..

[30] Lin Y., Wang Y., Li P.F. (2022). PPARα: An emerging target of metabolic syndrome, neurodegenerative and cardiovascular diseases. Front. Endocrinol..

